# On the Achievable Rate Region of the *K*-Receiver Broadcast Channels via Exhaustive Message Splitting

**DOI:** 10.3390/e23111408

**Published:** 2021-10-26

**Authors:** Rui Tang, Songjie Xie, Youlong Wu

**Affiliations:** 1 School of Information Science and Technology, ShanghaiTech University, Shanghai 201210, China; tangrui@shanghaitech.edu.cn (R.T.); xiesj@shanghaitech.edu.cn (S.X.); 2 Shanghai Institute of Microsystem and Information Technology, Chinese Academy of Sciences, Shanghai 200083, China; 3 University of Chinese Academy of Sciences, Beijing 100049, China

**Keywords:** broadcast channel, capacity region, covering lemma

## Abstract

This paper focuses on *K*-receiver discrete-time memoryless broadcast channels (DM-BCs) with private messages, where the transmitter wishes to convey *K* private messages to *K* receivers. A general inner bound on the capacity region is proposed based on an exhaustive message splitting and a *K*-level modified Marton’s coding. The key idea is to split every message into ∑j=1KKj−1 submessages each corresponding to a set of users who are assigned to recover them, and then send these submessages via codewords chosen from a *K*-level structure codebooks. To guarantee the joint typicality among all transmitted codewords, a sufficient condition on the subcodebooks’ sizes is derived through a newly establishing hierarchical covering lemma, which extends the 2-level multivariate covering lemma to the *K*-level case with more intricate dependences. As the number of auxiliary random variables and rate conditions both increase exponentially with *K*, the standard Fourier–Motzkin elimination procedure becomes infeasible when *K* is large. To tackle this problem, we obtain a *closed form* of achievable rate region with a special observation of disjoint unions of sets that constitute the power set of {1,⋯,K}. The proposed achievable rate region allows arbitrary input probability mass functions and improves over previously known achievable (closed form) rate regions for *K*-receiver (K≥3) BCs.

## 1. Introduction

The 2-receiver discrete-time memoryless broadcast channel (DM-BCs) was first introduced by Cover [1], who proposed the prestigious superposition coding that outperforms the traditional time-division strategy. Superposition coding is optimal for certain classes of BCs such as degraded DM-BCs, less noisy DM-BCs, and more capable DM-BCs [2,3]. The best known inner bound on the capacity region of DM-BCs is achieved by Marton’s coding with message splitting [4,5]. The key idea is to split each source message into common and private parts, where the common part is encoded into a cloud-center codeword, and two private parts are encoded into two separate codewords. To enlarge the achievable rate region, the submitted codewords are jointly typical which is guaranteed by a sufficient condition on the sizes of subcodebooks established by the covering lemma [2].

Recently, Gohari [6] improved the outer bounds for two-receiver broadcast channel by introducing an auxiliary receiver to derive the form of the bounds. He also improved Marton’s inner bounds on the cardinalities of the auxiliary variables [7]. For a non-asymptotic view, there are also improvements on the maximum coding rate based on a non-asymptotic converse and achievability bounds with feedback [8] on a two-user broadcast channel and also those bounds on the same coding rate of the common-message *K*-user DM-BC [9] which extends and strengthen the ones in [10].

There have been efforts on general capacity region without common broadcasting on Gaussian MIMO BCs [11] which coincides with dirty-paper coding (DPC) rate region. The capacity regions of physically degraded Gaussian BCs were further studied for the ergodic or composite situations [12,13,14]. However, the capacity region with a general message set for multi-antenna Gaussian broadcast channels is not known to full extent [15,16,17,18].

For the *K*-receiver (K≥2) DM-BCs, most previous works are based either on superposition coding (see in [19,20,21]) which requires certain constraints on Markov chains for auxiliary random variables (RVs), or on the 2-level Marton’s coding where each message is split into one common part and one private part [2,22]. The capacity region of broadcast channels of three receivers was studied with degraded message sets [23,24]. The capacity region of less-noisy broadcast channels with three receivers was determined [25], and superposition coding was justified to be sub-optimal for the more capable 3-receiver broadcast channels [26]. For arbitrary *K* case, the authors of [21] gave a general inner bound based on superposition coding and rate-splitting using notions from order theory and lattices wherein each receiver decodes its intended message (common or private) along with the partial interference designated to it through rate-splitting. The Marton’s coding with 2-level superposition coding structure (consisting of one cloud-center codebook and *K* satellite codebooks) can be easily constructed and analyzed by the multivariate covering lemma and packing lemma [2]. In [27], an evolved Marton’s coding scheme based on up-set [28] rate-splitting, superposition coding, and binning was introduced. However, this work failed to to derive a *close-form* achievable rate region due to the prohibitive complexity of applying the Fourier-Motzkin elimination procedure.

In this paper, we consider the general *K*-receiver (K≥2) DM-BCs with private messages. For the case K≥3, in order to inherit the characteristics of superposition coding and combine them with Marton’s coding, a general inner bound is proposed based on the exhaustive message splitting and a *K*-level modified Marton’s coding. More specifically, every message is split into ∑j=1KKj−1 submessages, with each corresponding to a set S belonging to the power set of {1,…,K}. The submessages respecting to S are encoded into an exclusive codeword and will be decoded by receiver *j* if j∈S. We enlarge the subcodebooks sizes and use a *K*-level Marton’s coding to send all codewords that are jointly typical with each other. This allows arbitrary dependence among the input RVs, rather than satisfying certain Markov chains as in [19,20,21,22]. Based on a newly established hierarchical covering lemma and properties of rate inequalities obtained in the scheme analysis, we obtain a closed-form achievable rate region. To the best of our knowledge, this is the first result of closed-form achievable rate region for general *K*-receiver DM-BCs allowing arbitrary input probability mass functions (pmfs). The proposed achievable rate region improves over all previously known closed-form achievable rate regions for *K*-receiver (K≥3) BCs.

Note that there are mainly two challenges on establishing the closed-form achievable rate region. The first one is how to derive rate conditions such that all transmitted codewords are jointly typical. The solution is related to the covering lemma. Unfortunately, the known multivariate covering lemma in [2] only deals with sequences which are conditionally independent, making it infeasible in our scheme since our inner bound allows arbitrary input pmfs. Additionally, the *recursive* mutual covering lemma in [27] imposes too many intertwined rate constraints, making it prohibitive to apply the Fourier–Motzkin elimination. To overcome these obstacles, we divide all RVs into *K* levels and process them hierarchically, leading to a new lemma called hierarchical covering lemma. With this lemma, we can keep joint typicality among all selected sequences, while reducing the number of rate conditions compared to the recursive covering lemma in [27]. The second challenge lies on how to apply Fourier–Motzkin elimination procedure to obtain the final form of the inner bound. Even with the newly hierarchical covering lemma, the number of rate conditions incurred by the packing lemma still increases exponentially with *K*. To tackle this problem, we aggregate the rate of submessages based on special observations of disjoint unions of sets that constitute the power set of {1,…,K}, and finally establish the final form of achievable rate region.

### Notations

Throughout the paper, we use the notation in [2]. In particular, we let

K≜{1,…,K}

for a positive integer *K*. We use P(K) to denote the power set of K, e.g., when K=3, P(K)={{1},{2},{3},{1,2},{1,3},{2,3},{1,2,3}}.

For any non-empty set $T=(k_{1},k_{2},\ldots ,k_{\left|T\right|})$, where $k_{i}\in K$ for i∈[1:|T|], denote $\Pi_{T}$ as the set including all permutations on T, i.e., $\Pi_{T}≜\{\left(\pi \left(1\right),\pi \left(2\right),\ldots ,\pi \left(\left|T\right|\right)\right):\pi \left(j\right)\in T,\pi \left(j\right)\neq \pi \left(i\right),\forall i,j\in [1:|T|],i\neq j\}$. We use ⨆ to denote the disjoint union, e.g., $T_{1}⨆T_{2}$ means $T_{1}\cup T_{2}$ with $T_{1}\cap T_{2}=\emptyset$.

Given a set $S=\left\{S_{1},S_{2},\ldots ,S_{\left|S\right|}\right\}$ with $S_{j}\subseteq K,\;\forall j\in \left[1:|S|\right]$, the subtuple of RVs with indices from S is denoted by $X\left(S\right)≜\left(X_{S_{1}},\ldots ,X_{S_{\left|S\right|}}\right)$, and the corresponding realizations by $x\left(S\right)≜\left(x_{S_{1}},\ldots ,x_{S_{\left|S\right|}}\right)$. Let $X^{n}\left(S\right)$ represent $\left(X_{S_{j}}^{n}:S_{j}\in S\right)$. For positive integers *k* and *j*, we define $x_{k}^{j}≜(x_{k,1},$ $\ldots ,x_{k,j})$, and $X_{k}^{j}≜\left(X_{k,1},\ldots ,X_{k,j}\right)$.

We use δ(ϵ)>0 to denote a function of ϵ that tends to zero as ϵ→0. When there are multiple functions $\delta_{1}\left(\epsilon \right),\ldots ,\delta_{k}\left(\epsilon \right)$, we denote them all by a generic function δ(ϵ) with the understanding that $\delta \left(\epsilon \right)=\max\{\delta_{1}\left(\epsilon \right),\ldots ,\delta_{k}\left(\epsilon \right)\}$.

## 2. Channel Model

**Figure 1:**
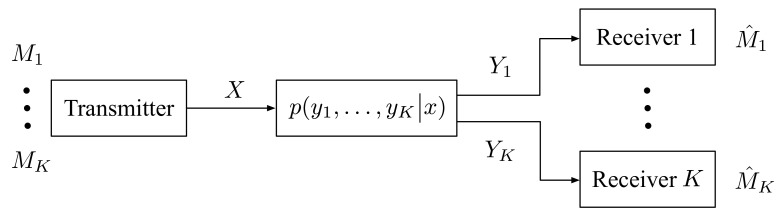
Broadcast channel for *K* receivers.

Consider a *K*-receiver DM-BC with only private messages depicted in Figure 1. The setup is characterized by a input alphabet X, *K* output alphabets $\left(Y_{k}:k\in K\right)$, and a collection of channel transition pmfs $p\left(y_{1},\cdots ,y_{K}|x\right)$. At time i∈[1:n], the transmitter sends the channel input $x_{i}\in X$, receiver k∈K observes the output $y_{k,i}\in Y_{k}$.

The goal of the communication is that the transmitter conveys private messages $M_{k}$ to receiver *k*, for k∈K, respectively. Each $M_{k}$ is independently and uniformly distributed over the set $M_{k}≜\left[1:2^{nR_{k}}\right]$, where $R_{k}$ denotes the communication rate of receiver *k*.

The encoder maps the messages $\left(M_{1},M_{2},\ldots ,M_{K}\right)$ to a sequence $x_{i}\in X$:

(1) $$X_{i}=f^{\left(n\right)}\left(M_{1},M_{2},\ldots ,M_{K}\right),$$

and receiver k∈K uses channel outputs $y_{k}^{n}$ to estimate ${\hat{M}}_{k}$ as a guess of messages $M_{k}$:

(2) $${\hat{M}}_{k}=g_{k}^{\left(n\right)}\left(Y_{k}^{n}\right).$$

A rate tuple $\left(R_{k}:k\in K\right)$ is called achievable if there exists an encoding function $f^{\left(n\right)}$ and *K* decoding functions $g_{1}^{\left(n\right)},\ldots ,g_{K}^{\left(n\right)}$ such that the error probability

(3) $$P_{e}^{\left(n\right)}=\mathrm{Pr}\left\{{\hat{M}}_{k}\neq M_{k},\exists \;k\in K\right\}$$

tends to zero as the block length *n* tends to infinity. The closure of the set of achievable rate tuple $\left(R_{k}:k\in K\right)$ is called the *capacity region*.

## 3. Preliminary

In this section, we present decomposition of sets which will be used to in Section 4 and Section 5.

Given a set S, an element k∈S and *l*, $l^{\prime }\in K$ with $l\leq l^{\prime }$, define

(4) $$\begin{array}{c} A_{S}^{l:l^{\prime }}\left(T\right)≜\left\{I\subseteq T:I⊇S,|I|=l,l+1,\cdots ,l^{\prime }\right\}, \end{array}$$

where $A_{S}^{l:l^{\prime }}\left(T\right)$ represents all subsets belonging to T, containing S and with cardinality between *l* and $l^{\prime }$. For simplicity, we omit the T, T and $l^{\prime }$ in $A_{S}^{l:l^{\prime }}\left(T\right)$ if S=∅, T=K or $l^{\prime }=l$, i.e.,

$$\begin{array}{c} A^{l:l^{\prime }}≜A_{\emptyset }^{l:l^{\prime }}\left(K\right),\;A_{S}^{l:l^{\prime }}≜A_{S}^{l:l^{\prime }}\left(K\right),\;A^{l}\;≜\;A^{l:l}. \end{array}$$

Given a set T, write its permutation $\pi_{T}\in \Pi_{T}$ as $\pi_{T}=\left(\pi \left(1\right),\pi \left(2\right),\ldots ,\pi (|T|)\right)$, where π(i) denotes the *i*-th element in $\pi_{T}$ (i.e., π(i)∈T), and π(i)≠π(k), if i≠k for all i,k∈[|T|]. Let $B_{\pi (i)}$ represent all subsets belonging to K\{π(1),…,π(i−1)} and containing π(i) with cardinality between *l* and $l^{\prime }$, i.e.,

(5) $$\begin{array}{c} B_{\pi (i)}^{l:l^{\prime }}≜A_{\left\{\pi (i)\right\}}^{l:l^{\prime }}\left(K\backslash \{\pi (1),\ldots ,\pi (i-1)\}\right). \end{array}$$

In particular, when i=1, we have

(6) $$\begin{array}{c} B_{\pi (1)}^{l:l^{\prime }}=A_{\left\{\pi (1)\right\}}^{l:l^{\prime }}. \end{array}$$

Similarly, we omit the $l^{\prime }$ if $l^{\prime }=l$, i.e., $B_{\pi (i)}^{l}≜\;B_{\pi (i)}^{l:l}.$ It is easy to show that $\{B_{\pi (i)}^{l:l^{\prime }}:i\in \left[|T|]\right\}$ are disjoint sets for any $l,l^{\prime }\in \left[1:K\right]$ and any subset T⊆K.

Next, we introduce the following lemma on decomposition among sets.

**Lemma** **1.** *The following decomposition holds:*

(7) $$\begin{array}{c} \underset{k\in T}{⋃}A_{\left\{k\right\}}^{l:l^{\prime }}=\overset{\left|T\right|}{\underset{i=1}{⨆}}B_{\pi (i)}^{l:l^{\prime }} \end{array}$$

**Proof.**  Note that

(8) $$\begin{array}{lll} \underset{k\in T}{⋃}A_{\left\{k\right\}}^{l:l^{\prime }} & = & \underset{k\in T}{⋃}\left\{I\subseteq T:I⊇\left\{k\right\},|I|=l,l+1,\cdots ,l^{\prime }\right\}\\ & = & \left\{I\subseteq K:I\cap T\neq \emptyset ,|I|=1,\ldots ,K\right\} \end{array}$$

(9) $$\begin{array}{rrr} & = & A_{\left\{\pi (1)\right\}}^{l:l^{\prime }}\cup A_{\left\{\pi (2)\right\}}^{l:l^{\prime }}\left(K\backslash \left\{\pi \left(1\right)\right\}\right)\cup \cdots \cup A_{\left\{\pi (|T|)\right\}}^{l:l^{\prime }}\left(K\backslash \{\pi (1),\ldots ,\pi (|T|-1)\}\right)\\ & = & \overset{\left|T\right|}{\underset{i=1}{⨆}}B_{\pi (i)}^{l:l^{\prime }}, \end{array}$$

where the last equality holds by (5) and as $\{B_{\pi (i)}^{l:l^{\prime }}:i\in \left[|T|]\right\}$ are disjoint sets. □

For example, consider the case K=3, T=(π(1),π(2),π(3))={1,2,3} and $\pi_{T}=\left(1,2,3\right)$, we have

$$\begin{array}{ccc} A_{\left\{1\right\}}^{1:2}\left(\left\{1,2,3\right\}\right) & = & \{\{1\},\{1,2\},\{1,3\}\},\\ \underset{k\in T}{⋃}A_{\left\{k\right\}}^{1:K} & = & \{\{1\},\{2,3\},\{1,2\},\{1,3\},\{2,3\},\{1,2,3\}\},\\ B_{\pi (1)}^{1:3} & = & \left\{\left\{1\right\},\left\{1,2\right\},\left\{1,3\right\},\left\{1,2,3\right\}\right\},\;B_{\pi (2)}^{1:3}=\left\{\left\{2\right\},\left\{2,3\right\}\right\},\;B_{\pi (3)}^{1:3}=\left\{3\right\}. \end{array}$$

**Figure 2:**
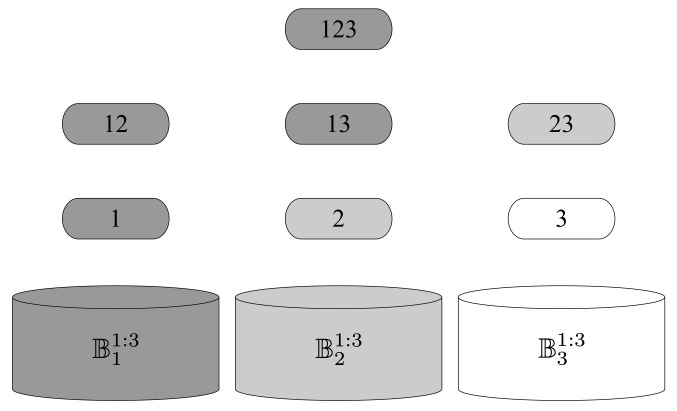
The illustration of the disjoint decomposition of ${⋃}_{k\in T}A_{\left\{k\right\}}^{1:K}$ in the case for K=3, T={1,2,3} and π=(π(1),π(2),π(3))=(1,2,3). The idea is that every time we pick a collection of sets descended from a set in the layer *l*, the number of unpicked sets in layer l−1 is one, where layer *l* contains all subsets of K with cardinality *l*. Therefore, ${⋃}_{k\in [1:3]}A_{\left\{k\right\}}^{1:3}=B_{1}^{1:3}⨆B_{2}^{1:3}⨆B_{3}^{1:3}$.

Figure 2 is given for illustration. To simplify the notation, an arbitrary collection of sets, e.g., {1,12,123}, is recognized as {{1},{1,2},{1,2,3}}

## 4. Main Results

**Theorem** **1.** *Using the notations presented in Section 3, a rate tuple $\left(R_{k}:k\in K\right)$ is achievable for the DM-BC $p\left(y_{1},\ldots ,y_{K}|x\right)$ if for all T⊆K and permutations $\pi_{T}=\left\{\pi \left(1\right),\ldots ,\pi (|T|)\right\}\in \Pi_{T}$,*

(10) $$\begin{array}{c} \sum_{k\in T}R_{k}\leq \sum_{i=1}^{\left|T\right|}(\sum_{S\in B_{\pi (i)}^{1:K}}H\left(U_{S}|U\left(A_{S}^{1:K}\backslash S\right)\;-\;H\left(U\left(B_{\pi (i)}^{1:K}\right)|Y_{\pi (i)}U\left(A_{\pi (i)}^{1:K}\backslash B_{\pi (i)}^{1:K}\right)\right)\right)\\ \;+\sum_{l=1}^{K-1}\left(H\left(U\left(\overset{\left|T\right|}{\underset{i=1}{⨆}}B_{\pi (i)}^{l}\right)|U\left(A^{(l+1):K}\right)\right)-\sum_{S\in {⨆}_{i=1}^{\left|T\right|}B_{\pi (i)}^{l}}H\left(U_{S}|U\left(A_{S}^{1:K}\backslash S\right)\right)\right), \end{array}$$

*for some pmf $p(U(A^{1:K}))$ and a function $X=f(U\left(A^{1:K}\right))$.*

**Proof.**  The achievable scheme is based on an exhaustive message splitting and a K-level Marton’s coding. More specifically, each message $M_{k}$, for k∈K, is split into ∑j=1KKj−1 submessages $M_{k}≜\left(M_{k,S}:S\subseteq K,\;\left|S\right|=1,2,\cdots ,K\right)$ with $M_{k,S}\in \emptyset$ if k∉S. The submessages $M_{S}=\left(M_{k,S}:k\in K\right)$ are encoded into a codeword $u_{S}^{n}∼p\left(u_{S}|u\left(A_{S}^{1:K}\backslash S\right)\right)$ and will be decoded by receiver *j* if j∈S. Then, a *K*-level Marton’s coding is applied to send $\left(2^{K}-1\right)$ codewords $\left(u_{S}^{n}:S\subseteq K,|S|=1,\ldots ,K\right)$ that are jointly typical with each other. See detailed scheme in Section 5. □

To ensure arbitrary input pmfs $p(U(A^{1:K}))$ in the inner bound above, we need to establish a sufficient condition on the sizes of subcodebooks, which is related to the covering lemma. The multivariate covering lemma in [2] only consider sequences generated under simple dependence, e.g., $\left(u_{1}^{n},\ldots ,u_{K}^{n}\right)∼p\left(u_{1}|u_{0}\right)p\left(u_{1}|u_{0}\right)\ldots p\left(u_{K}|u_{0}\right)$ given a sequence $u_{0}^{n}∼P_{U_{0}}$. In our scheme there are $\left(2^{K}-1\right)$ RVs, represented by $\left(U_{S}:S\in U\left(A^{1:K}\right)\right)$, and each symbol of codeword $U_{S}^{n}$ is generated conditionally independent according to $p(u_{S}|u\left(A_{S}^{1:K}\backslash S\right))$. In [27], a recursive mutual covering Lemma was introduced, which allows all transmitted codewords to be jointly typical. However, the recursive mutual covering lemma creates around $2^{K}$ intertwined rate equalities, turning the Fourier–Motzkin elimination procedure to a disastrous problem. To obtain the closed-form achievable rate region, we divide subcodebooks into *K* levels and process them recursively in a hierarchical manner, which greatly reduces the number of rate inequalities and enables to obtain the closed-form rate region.

**Lemma** **2** (Hierarchical Covering Lemma)**.** *Let $U\left(A^{1:K}\right)∼p\left(u\left(A^{1:K}\right)\right)$ and $\epsilon_{K}<\epsilon_{K-1}\cdots <\epsilon_{1}$. Let $U_{K}^{n}∼p\left(u_{K}^{n}\right)$ be a random sequence with $\lim_{n\to \infty }P\{\;U_{K}^{n}\in T_{\epsilon_{K}}^{\left(n\right)}\;\}=1$. For each l∈[1:K−1] and $S\in A^{l}$, let $U_{S}^{n}\left(m_{S}\right)$, $m_{S}\in \left[1:2^{nr_{S}}\right]$, be pairwise conditionally independent sequences, each distributed according to $\prod_{i=1}^{n}p_{U_{S}|U\left(A_{S}^{1:K}\backslash S\right)}\left(u_{S,i}|u_{S^{\prime },i}:S^{\prime }\in \left(A_{S}^{1:K}\backslash S\right)\right)$. Assume that $\left(U_{S}^{n}\left(m_{S}\right):m_{S}\in \left[1:2^{nr_{S}}\right],\;S\in A^{l}\right)$ are mutually conditionally independent given $(U_{S}^{n}\left(m_{S}\right):S\in \left(A_{S}^{1:K}\backslash S\right)$. Then, there exists $\delta (\epsilon_{l})$ that tends to zero as $\epsilon_{l}\to$ 0 such that*

$$\begin{array}{cc} \; & \lim_{n\to \infty }P\{\left(U_{S}^{n}\left(m_{S}\right):S\in A^{1:(K-1)},U_{K}^{n}\right)\notin T_{\epsilon_{1}}^{\left(n\right)}\\ \; & \mathrm{for}\;\mathrm{all}\;\left(m_{S}:S\in A^{1:(K-1)}\right)\in \;\;\;\;\prod_{S\in A^{1:(K-1)}}\;\;\;\;\left[1:2^{nr_{S}}\right]\}=0, \end{array}$$

*if*

(11) $$\begin{array}{cc} \sum_{S\in J^{l}}r_{S}> & \sum_{S\in J^{l}}H\left(U_{S}|U\left(A_{S}^{1:K}\backslash S\right)\right)-H\left(U\left(J^{l}\right)|U\left(A^{(l+1):K}\right)\right)+\delta \left(\epsilon_{l}\right), \end{array}$$

*for all $J^{l}\subseteq A^{l}$ and l∈[1:(K−1)].*

**Proof.**  See the proof in Appendix A. □

**Remark** **1.** *The main difference between recursive mutual covering lemma [27] is that our hierarchical covering lemma reduces the number of variables (this is achieved by assuring the typicality of the codewords top-down from level K to level 1 in the proof of hierarchical covering lemma) in almost each inequalities compared with those in [27], which makes it easier for Fourier-Motzkin elimination to derive a closed-form achievable rate region. However, The accurate performance needs to be justified on specific channels, which also requires an optimal choice of the distribution of the auxiliary variables. Thus, it is hard to evaluate the overall performance, and we leave it in our future work.*

*To briefly investigate the performance of the two covering lemmas, we give an example of the bound on $r_{1}+r_{12}+r_{13}+r_{123}$ of each.*

*Utilizing our lemma, we get three inequalities to combine it. Namely,*

$$\begin{array}{ccc} r_{1} & & >H\left(U_{1}|U_{12}U_{13}U_{123}\right)-H\left(U_{1}|U_{12}U_{13}U_{23}U_{123}\right)\\ r_{12}+r_{13} & & >H\left(U_{12}|U_{123}\right)+H\left(U_{13}|U_{123}\right)-H\left(U_{12}U_{13}|U_{123}\right)\\ r_{123} & & >0. \end{array}$$

*By adding them all together, we get $r_{1}+r_{12}+r_{13}+r_{123}>H\left(U_{1}|U_{12}U_{13}U_{123}\right)+H\left(U_{12}|U_{123}\right)+H\left(U_{13}|U_{123}\right)-\left(H\left(U_{1}|U_{12}U_{13}U_{23}U_{123}\right)+H\left(U_{12}U_{13}|U_{123}\right)\right)$.*

*On the other hand, the bound on it by recursive mutual covering lemma is $H\left(U_{1}|U_{12}U_{13}U_{123}\right)+H\left(U_{12}|U_{123}\right)+H\left(U_{13}|U_{123}\right)-H\left(U_{1}U_{12}U_{13}|U_{123}\right)$, namely $H\left(U_{1}|U_{12}U_{13}U_{123}\right)+H\left(U_{12}|U_{123}\right)+H\left(U_{13}|U_{123}\right)-\left(H\left(U_{1}|U_{12}U_{13}U_{123}\right)+H\left(U_{12}U_{13}|U_{123}\right)\right)$, which leads to a lower upper bound (by conditioning reduces entropy) compared with ours, and our scheme may sacrifice some performance to derive a closed-form achievable rate tuple constraints.*

**Lemma** **3.** *Consider the inequality condition in the hierarchical covering lemma:*

$$\begin{array}{cc} \sum_{S\in J^{l}}r_{S}> & \sum_{S\in J^{l}}H\left(U_{S}|U\left(A_{S}^{1:K}\backslash S\right)\right)-H\left(U\left(J^{l}\right)|U\left(A^{(l+1):K}\right)\right)+\delta \left(\epsilon \right). \end{array}$$

*If the set $J^{l}$ is split into $N\in [\;1:|J^{l}|\;]$ disjoint pieces $\{J_{1}^{l},J_{2}^{l},\ldots ,J_{N}^{l}\}$ satisfying*

(12) $$\begin{array}{c} J^{l}=\overset{N}{\underset{i=1}{⋃}}J_{i}^{l},\;with\;J_{i}^{l}\cap J_{j}^{l}=\emptyset ,\;if\;i\neq j, \end{array}$$

*then for all i∈[N], we have*

$$\begin{array}{cc} \sum_{S\in J_{i}^{l}}r_{S}> & \sum_{S\in J_{i}^{l}}H\left(U_{S}|U\left(A_{S}^{1:K}\backslash S\right)\right)-H\left(U\left(J_{i}^{l}\right)|U\left(A^{(l+1):K}\right)\right)+\delta_{J_{i}^{l}}\left(\epsilon \right). \end{array}$$

*With simple justification, the sum of lower bounds for $\sum_{S\in J_{i}^{l}}r_{S},\;\forall i\in \left[N\right]$ is not greater than the lower bound for $\sum_{S\in J^{l}}r_{S}$, which means combinations of split inequalities will not induce a smaller region bounded by the overall inequalities that simultaneously restrain all variables,*

**Proof.**  It is equivalent to prove that

$$\begin{array}{ccc} & & \sum_{S\in J^{l}}H\left(U_{S}|U\left(A_{S}^{1:K}\backslash S\right)\right)-H\left(U\left(J^{l}\right)|U\left(A^{(l+1):K}\right)\right)\\ & & \overset{\left(a\right)}{=}\sum_{i=1}^{N}\sum_{S\in J_{i}^{l}}\left(H\left(U_{S}|U\left(A_{S}^{1:K}\backslash S\right)\right)-H\left(U\left(J_{i}^{l}\right)|U\left(A^{(l+1):K}\right),U\left(J_{1}^{l}\right),\ldots ,U\left(J_{i-1}^{l}\right)\right)\right)\\ & & \geq \sum_{i=1}^{N}\left(\sum_{S\in J_{i}^{l}}H\left(U_{S}|U\left(A_{S}^{1:K}\backslash S\right)\right)-H\left(U\left(J_{i}^{l}\right)|U\left(A^{(l+1):K}\right)\right)\right), \end{array}$$

where (a) follows by the chain rule of entropy and (12); (b) holds because conditioning reduces entropy. □

**Corollary** **1.** *Define*

$$\begin{array}{c} I_{12}:=\min_{i\in \{1,2\}}\left\{I\left(U_{12}U_{123};Y_{i}\right)\right\},\;I_{13}:=\min_{i\in \{1,3\}}\left\{I\left(U_{13}U_{123};Y_{i}\right)\right\},\;I_{23}:=\min_{i\in \{2,3\}}\left\{I\left(U_{23}U_{123};Y_{i}\right)\right\}, \end{array}$$

*and*

$$\begin{array}{ccc} \Delta & := & I\left(U_{2};U_{13}|U_{12}U_{23}U_{123}\right)+I\left(U_{3};U_{12}|U_{13}U_{23}U_{123}\right)+I\left(U_{1};U_{3}|U_{2}U_{12}U_{13}U_{23}U_{123}\right)\\ & & +I\left(U_{1};U_{23}|U_{12}U_{13}U_{123}\right)+I\left(U_{1};U_{2}|U_{12}U_{13}U_{23}U_{123}\right)+I\left(U_{2};U_{3}|U_{12}U_{13}U_{23}U_{123}\right). \end{array}$$

*A rate region $\left(R_{1},R_{2},R_{3}\right)$ is achievable for 3-receiver DM-BC $p(y_{1},y_{2},y_{3}|x)$ if*

(13a) $$\begin{array}{lll} R_{1}\; & < & I\left(U_{1}U_{12}U_{13}U_{123};Y_{1}\right)-I\left(U_{1};U_{23}|U_{12}U_{13}U_{123}\right),\; \end{array}$$

(13b) $$\begin{array}{lll} R_{2}\; & < & I\left(U_{2}U_{12}U_{23}U_{123};Y_{2}\right)-I\left(U_{2};U_{13}|U_{12}U_{23}U_{123}\right), \end{array}$$

(13c) $$\begin{array}{lll} R_{3}\; & < & I\left(U_{3}U_{13}U_{23}U_{123};Y_{3}\right)-I\left(U_{3};U_{12}|U_{13}U_{23}U_{123}\right),\;\; \end{array}$$

(13d) $$\begin{array}{lll} R_{1}\;+\;R_{2} & < & I_{12}+I\left(U_{1}U_{13};Y_{1}|U_{12}U_{123}\right)+I\left(U_{2}U_{23};Y_{2}|U_{12}U_{123}\right)\\ \; & \; & \;\;-I\left(U_{2};U_{13}|U_{13}U_{23}U_{123}\right)\\ \; & \; & \;\;-I\left(U_{13};U_{23}|U_{12}U_{123}\right)\\ \; & \; & \;\;-I\left(U_{1};U_{23}|U_{12}U_{13}U_{123}\right)-I\left(U_{1};U_{2}|U_{12}U_{13}U_{23}U_{123}\right), \end{array}$$

(13e) $$\begin{array}{lll} R_{1}\;+\;R_{3} & < & I_{13}+I\left(U_{1}U_{12};Y_{1}|U_{13}U_{123}\right)+I\left(U_{3}U_{23};Y_{3}|U_{13}U_{123}\right)\\ \; & \; & \;\;-I\left(U_{12};U_{23}|U_{13}U_{123}\right)-I\left(U_{3};U_{12}\right|U_{13}U_{23}U_{123}\\ \; & \; & \;\;-I\left(U_{1};U_{23}|U_{12}U_{13}U_{123}\right)-I\left(U_{1};U_{3}|U_{12}U_{13}U_{23}U_{123}\right)), \end{array}$$

(13f) $$\begin{array}{lll} R_{2}\;+\;R_{3} & < & I_{23}+I\left(U_{2}U_{12};Y_{2}|U_{23}U_{123}\right)+I\left(U_{3}U_{13};Y_{3}|U_{23}U_{123}\right)\\ \; & \; & \;\;-I\left(U_{12};U_{13}|U_{23}U_{123}\right)-I\left(U_{3};U_{12}|U_{13}U_{23}U_{123}\right)\\ \; & \; & \;\;-I\left(U_{2};U_{13}|U_{12}U_{13}U_{123}\right)-I\left(U_{2};U_{3}|U_{12}U_{13}U_{23}U_{123}\right), \end{array}$$

(13g) $$\begin{array}{lll} R_{1}\;+\;R_{2}+R_{3} & < & I_{12}+I\left(U_{1}U_{13};Y_{1}|U_{12}U_{123}\right)+I\left(U_{2}U_{23};Y_{2}|U_{12}U_{123}\right)\\ \; & \; & \;\;+I\left(U_{3};Y_{3}|U_{13}U_{23}U_{123}\right)-I\left(U_{13}U_{23}|U_{12}U_{123}\right)-\Delta , \end{array}$$

(13h) $$\begin{array}{lll} R_{1}\;+\;R_{2}+R_{3} & < & I_{13}+I\left(U_{1}U_{12};Y_{1}|U_{13}U_{123}\right)+I\left(U_{3}U_{23};Y_{3}|U_{13}U_{123}\right)\\ \; & \; & \;\;+I\left(U_{2};Y_{2}|U_{12}U_{23}U_{123}\right)-I\left(U_{12}U_{23}|U_{13}U_{123}\right)-\Delta , \end{array}$$

(13i) $$\begin{array}{lll} R_{1}\;+\;R_{2}+R_{3} & < & I_{23}+I\left(U_{2}U_{12};Y_{2}|U_{23}U_{123}\right)+I\left(U_{3}U_{13};Y_{3}|U_{23}U_{123}\right)\\ \; & \; & \;\;+I\left(U_{1};Y_{1}|U_{12}U_{13}U_{123}\right)-I\left(U_{12}U_{13}|U_{23}U_{123}\right)-\Delta , \end{array}$$

*for some pmf $p(u_{1}u_{2}u_{3}u_{12}u_{13}u_{23}u_{123})$ and a function $x=f(u_{1}u_{2}u_{3}u_{12}u_{13}u_{23}u_{123})$.*

**Proof.**  The result directly comes from Theorem 1 with K=3. For example, for T={1,2} and $\pi_{T}=\left(1,2\right)$, in (10), then we have $A^{3}=\left\{123\right\}$, $A^{2:3}=\left\{12,13,23,123\right\}$, and,

$$\begin{array}{ccc} B_{\pi (1)}^{1:3} & = & \left\{123,12,13,1\right\},\;B_{\pi (2)}^{1:3}=\left\{23,2\right\}, \end{array}$$

which satisfy the decomposition $\cup_{i\in \{1,2\}}A_{\left\{i\right\}}^{1:3}=\left\{1,12,13,23,123\right\}=B_{\pi (1)}^{1:3}⊔B_{\pi (2)}^{1:3}$. With $\pi_{T}=\left(1,2\right)$ and $\pi_{T}=\left(2,1\right)$, we obtain upper bound for $R_{1}+R_{2}$ in (13d). The remaining rate constraints are acquired similarly. □

**Remark** **2** (Comparison with superposition coding and Marton’s coding)**.** *Our achievable region generalizes that introduced by standard Marton’s coding and superposition coding, i.e., both of them are special cases of our general-form intended coding. Furthermore, the rate region in Theorem 1 contains the regions resulted from the two aforementioned coding schemes.*

- *Our coding degenerates into Marton’s coding by setting $U_{S}=const$, $\forall S\in A^{2:(K-1)}$, which indicates our rate region contains that derived by Marton’s coding;*
- *As superposition coding is optimal for degraded/less noisy/more capable DM-BC, which is barely guaranteed in many cases, i.e., without knowing the concrete relation according to the Markov chain, our rate region contains that derived from superposition coding.*

*In the future work, we will evaluate our rate region for some specific DM-BCs to show that our coding scheme strictly improves previously known inner bounds.*

## 5. Achievable Coding Scheme for Theorem 1

We first present our scheme for 3-receiver DM-BC as an illustration, and then extend it to general *K*-receiver DM-BC model for K≥2.

### 5.1. Coding Scheme for 3-Receiver DM-BCs

Let an all-one column vector (1,…,1) with a specified dimension be denoted by **1**. Albeit with an abuse of notation, we use a string of elements to implify the singleton notations in Section 3, e.g., $R_{123}=R_{\left\{123\right\}}$, $M_{123}=M_{\left\{123\right\}}$, $A_{123}^{l:l^{\prime }}=A_{\left\{123\right\}}^{l:l^{\prime }}$, $l_{123}=l_{\left\{123\right\}}$ etc. To simplify notations, we denote $U_{S}^{n}\left(m_{S},l_{S}|m_{A_{S}^{1:K}\backslash S},l_{A_{S}^{1:K}\backslash S}\right)$ by $U_{S}^{n}\left(m_{S},l_{S}\right)$ and its realization $u_{S}^{n}\left(m_{S},l_{S}|m_{A_{S}^{1:K}\backslash S},l_{A_{S}^{1:K}\backslash S}\right)$ by $u_{S}^{n}\left(m_{S},l_{S}\right)$.

#### 5.1.1. Rate Splitting

Divide $M_{j}\in \left[1:2^{R_{j}}\right]$, j∈{1,2,3}, into four independent messages $(M_{j,S}\in \left[1:2^{R_{j,S}}\right]$: $S\in A_{\left\{j\right\}}^{1:3})$. Therefore, $R_{j}=\sum_{S\in A_{\left\{j\right\}}^{1:3}}R_{j,S}$. More precisely,

$$\begin{array}{cc} M_{1} & =(M_{1,123},M_{1,12},M_{1,13},M_{1,1}),\\ M_{2} & =(M_{2,123},M_{2,12},M_{2,23},M_{2,2}),\\ M_{3} & =(M_{3,123},M_{3,23},M_{3,13},M_{3,3}),\\ R_{1} & =R_{1,123}+R_{1,12}+R_{1,13}+R_{1,1},\\ R_{2} & =R_{2,123}+R_{2,12}+R_{2,23}+R_{2,2},\\ R_{3} & =R_{3,123}+R_{3,23}+R_{3,13}+R_{3,3}. \end{array}$$

For convenience, let $R_{123}≜R_{1,123}+R_{2,123}+R_{3,123}$, $R_{12}≜R_{1,12}+R_{2,12}$, $R_{13}≜R_{1,13}+R_{3,13}$, $R_{23}≜R_{2,23}+R_{3,23}$, $R_{1}≜R_{1,1}$, $R_{2}≜R_{2,2}$, $R_{3}≜R_{3,3}$ and $M_{123}≜\left(M_{1,123},M_{2,123},M_{3,123}\right)$, $M_{12}≜\left(M_{1,12},M_{2,12}\right)$, $M_{23}≜\left(M_{2,23},M_{3,23}\right)$, $M_{13}≜\left(M_{1,13},M_{3,13}\right)$, $M_{1}≜M_{1,1}$, $M_{2}≜M_{2,2}$, $M_{3}≜M_{3,3}$.

#### 5.1.2. Codebook Generation

Fix a pmf $p(u_{123},u_{12},u_{23},u_{13},$ $u_{1},u_{2},u_{3})$. Randomly and independently generate $2^{nR_{123}}$ sequences $u_{123}^{n}\left(m\right)$ each according to $\prod_{i=1}^{n}p_{U_{123}}\left(u_{123,i}\right)$. For each $m_{12}\in \left[1:2^{nR_{1,12}}\right]\times \left[1:2^{nR_{2,12}}\right]\times \left[1:2^{nR_{3,12}}\right]$ generate a subcodebook $C_{12}\left(m_{12}\right)$ consisting of $2^{n({\tilde{R}}_{12}-R_{12})}$ independent generated sequences $u_{12}^{n}\left(m_{12},l_{12}\right)$, $l_{12}\in \left[1:2^{n({\tilde{R}}_{12}-R_{12})}\right]$ , each according to $\prod_{i=1}^{n}p_{U_{12}|U_{123}}\left(u_{12,i}|u_{123,i}\right)$. In the same way, for each $m_{23}$ and $m_{13}$, generate subcodebooks $C_{23}\left(m_{23}\right)$ and $C_{13}\left(m_{13}\right)$, respectively. For each $m_{1}\in \left[1:2^{nR_{1,1}}\right]$, generate a subcodebook $C_{1}\left(m_{1}\right)$ consisting of $2^{n({\tilde{R}}_{1}-R_{1})}$ independent generated sequences $u_{1}^{n}\left(m_{1},l_{1}\right)$, $l_{1}\in \left[1:2^{n({\tilde{R}}_{1}-R_{1})}\right]$ , each according to $\prod_{i=1}^{n}p_{U_{1}|U_{12}U_{13}U_{123}}\left(u_{1,i}|u_{12,i}u_{13,i}u_{123,i}\right)$. For $m_{2}$ and $m_{3}$, generate corresponding subcodebooks similarly.

#### 5.1.3. Encoding

For each $m_{S}$, S∈{1,2,3,12,13,23}, find an index tuple $\left(l_{1},l_{2},l_{3},l_{12},l_{13},l_{23}\right)$ such that $u_{S}^{n}\left(m_{S},l_{S}\right)\in C_{S}\left(m_{S}\right),S\in \left\{1,2,3,12,13,23\right\}$, and

(14) $$\begin{array}{c} \left(u_{S}^{n}\left(m_{S},l_{S}\right):S\in \left\{1,2,3,12,13,23\right\}\right)\in T_{\epsilon^{\prime }}^{\left(n\right)} \end{array}$$

If more than one such index tuple can be found, choose an arbitrary one among those. If no such tuple exists, just choose $\left(l_{1},l_{2},l_{3},l_{12},l_{13},l_{23}\right)=\left(1,1,1,1,1,1\right)$. Then, the transmitter generates $x^{n}\left(m_{123},m_{12},m_{13},m_{23},m_{1,1},m_{2,2},m_{3,3}\right)$ as

$$\begin{array}{cc} x_{i}=x( & u_{123,i}\left(m_{123}\right),u_{12,i}\left(m_{12},l_{123}\right),u_{13,i}\left(m_{13},l_{13}\right),u_{23,i}\left(m_{23},l_{23}\right),\\ \; & u_{1,i}\left(m_{1},l_{1}\right),u_{2,i}\left(m_{2},l_{2}\right)),u_{3,i}\left(m_{3},l_{3}\right))),\;\mathrm{for}\;i=1,\ldots ,n. \end{array}$$

#### 5.1.4. Decoding

Decoder j=1 declares that $({\hat{m}}_{1,123},{\hat{m}}_{1,12},$${\hat{m}}_{1,13},{\hat{m}}_{1,1})$ is sent if it is the unique message such that

$$\begin{array}{c} \left(u_{123}^{n}\left(\hat{m_{123}}\right),u_{S}^{n}\left({\hat{m}}_{S},l_{S}\right):S\in \left\{1,12,13\right\},y_{1}^{n}\right)\in T_{\epsilon }^{\left(n\right)}. \end{array}$$

Similarly, receiver j=2,3 uses joint typicality decoding to find the unique message $\left({\hat{m}}_{2,123},{\hat{m}}_{2,12},{\hat{m}}_{2,23},{\hat{m}}_{2,2}\right)$ and $\left({\hat{m}}_{3,123},{\hat{m}}_{3,13},{\hat{m}}_{3,23},{\hat{m}}_{3,3}\right)$, respectively.

#### 5.1.5. Analysis of the Probability of Error

Assume without loss of generality that

$$\begin{array}{c} \left(M_{123},M_{12},M_{13},M_{23},M_{1},M_{2},M_{3}\right)=\left(1,1,1,1,1,1,1\right) \end{array}$$

is sent and let $L_{12},L_{13},L_{23},L_{1},L_{2},L_{3}$ be the index tuple of selected sequences. We note that the subcodebook $C_{S}\left(m_{S}\right)$ consists of $2^{n({\tilde{R}}_{S}-R_{S})}$ i.i.d. $U_{S}^{n}\left(m_{S},l_{S}\right)$ sequences, ∀S∈{1,2,3,12,13,23}. By the hierarchical covering lemma (with $r_{S}={\tilde{R}}_{S}-R_{S}$), we obtain a set of constraints for S∈{12,13,23}

(15a)r12+r13>I(U12;U13|U123),(15b)r12+r23>I(U12;U23|U123),(15c)r13+r23>I(U13;U23|U123),(15d)r12+r13+r23>I(U12;U23|U123)+I(U13;U23|U123)+I(U12;U13|U123,U23).

Again, using hierarchical covering lemma for S∈{1,2,3}, we attain the region for $r_{1},r_{2},r_{3}$:

(16a)r1>I(U1;U23|U123U12U13),(16b)r2>I(U2;U13|U123U12U23),(16c)r3>I(U3;U12|U123U13U23),(16d)r1+r2>I(U1;U2|U123U12U13U23)+I(U1;U23|U123U12U13)+I(U2;U13|U123U23U12),(16e)r1+r3>I(U1;U3|U123U12U13U23)+I(U1;U23|U123U12U13)+I(U3;U12|U123U13U23),(16f)r2+r3>I(U2;U3|U123U12U13U23)+I(U2;U13|U123U12U23)+I(U3;U12|U123U13U23),r1+r2+r3>I(U1;U2|U123U12U23U13)+I(U2;U3|U123U12U13U23)+I(U3;U12|U123U13U23)(16g)+I(U1;U23|U123U12U13)+I(U2;U13|U123U23U12)+I(U1;U3|U123U12,U23U13U2).

By the symmetry of decoders, we first consider the average probability of error for decoder 1. To analyze it, the Table 1 list all possible pmfs of message tuple $\left(U_{123}^{n}\left(m_{123}\right),U_{12}^{n}\left(m_{12},l^{\prime }\right),U_{13}^{n}\left(m_{13},l^{\prime }\right),U_{1}^{n}\left(m_{1},l^{\prime }\right),Y_{1}^{n}\right)$.

$$\begin{array}{ccc} & & E_{1,0}=\left\{\left(U_{123}^{n}\left(1\right),U_{12}^{n}\left(1,l\right),U_{13}^{n}\left(1,l\right),U_{1}^{n}\left(1,l\right),Y_{1}^{n}\right)\notin T_{\epsilon }^{\left(n\right)}\right\},\\ & & E_{1,1}=\left\{\left(U_{123}^{n}\left(1\right),U_{12}^{n}\left(1,l\right),U_{13}^{n}\left(1,l\right),U_{1}^{n}\left(*\right),Y_{1}^{n}\right)\in T_{\epsilon }^{\left(n\right)}\right\},\\ & & E_{1,13}=\left\{\left(U_{123}^{n}\left(1\right),U_{12}^{n}\left(1,l\right),U_{13}^{n}\left(*\right),U_{1}^{n}\left(*\right),Y_{1}^{n}\right)\in T_{\epsilon }^{\left(n\right)}\right\},\\ & & E_{1,12}=\left\{\left(U_{123}^{n}\left(1\right),U_{12}^{n}\left(*\right),U_{13}^{n}\left(1,l\right),U_{1}^{n}\left(*\right),Y_{1}^{n}\right)\in T_{\epsilon }^{\left(n\right)}\right\},\\ & & E_{1,12,13}=\left\{\left(U_{123}^{n}\left(1\right),U_{12}^{n}\left(*\right),U_{13}^{n}\left(*\right),U_{1}^{n}\left(*\right),Y_{1}^{n}\right)\in T_{\epsilon }^{\left(n\right)}\right\},\\ & & E_{1,123}=\left\{\left(U_{123}^{n}\left(*\right),U_{12}^{n}\left(*\right),U_{13}^{n}\left(*\right),U_{1}^{n}\left(*\right),Y_{1}^{n}\right)\in T_{\epsilon }^{\left(n\right)}\right\}. \end{array}$$

By the conditional typicality lemma, the first term $P(E_{1,0})$ tends to zero as n→∞ as $\left(u_{S}^{n}\left(m_{S},l_{S}\right):S\in \left\{1,2,3,12,13,23\right\}\right)\in T_{\epsilon^{\prime }}^{\left(n\right)}$ and $\epsilon^{\prime }<\epsilon$.

Considering the second term $P(E_{1,1})$, note that $U_{1}^{n}\left(l_{1}\right)∼\prod_{i=1}^{n}P_{U_{1}}\left(u_{1,i}\right)$ is independent of $Y_{1}^{n}$ for every $U_{1}\left(l_{1}\right)\notin C_{1}\left(1\right)$, then the variables $U_{1}^{n}\left(*\right)$ is independent of $Y_{1}^{n}$ on condition of $\left(U_{123}^{n}\left(1\right),U_{12}^{n}\left(1,l_{12}\right),U_{13}^{n}\left(1,l_{13}\right)\right)$. By the packing lemma, $P(E_{1,1})$ tends to zero as n→∞ if

$$\begin{array}{cc} {\tilde{R}}_{1} & <I(U_{1};Y_{1}|U_{123}U_{12}U_{13}). \end{array}$$

Similarly, $P(E_{1,12})$, $P(E_{1,13})$, $P(E_{1,12,13})$ and $P(E_{1,123})$ tends to zero as n→∞ if

$$\begin{array}{cc} {\tilde{R}}_{12} & +{\tilde{R}}_{1}<I\left(U_{12}U_{1};Y_{1}|U_{123}U_{13}\right)+I\left(U_{12};U_{13}|U_{123}\right),\\ {\tilde{R}}_{13} & +{\tilde{R}}_{1}<I\left(U_{13}U_{1};Y_{1}|U_{123}U_{12}\right)+I\left(U_{12};U_{13}|U_{123}\right),\\ {\tilde{R}}_{12} & +{\tilde{R}}_{13}+{\tilde{R}}_{1}<I\left(U_{12}U_{13}U_{1};Y_{1}|U_{123}\right)+I\left(U_{12};U_{13}|U_{123}\right),\\ R_{123} & +{\tilde{R}}_{12}+{\tilde{R}}_{13}+{\tilde{R}}_{1}<I\left(U_{123}U_{12}U_{13}U_{1};Y_{1}\right)+I\left(U_{12};U_{13}|U_{123}\right). \end{array}$$

For receiver 2 and 3, the corresponding inequalities can be derived in a similar way.

From the lower bound of linear combinations of $r_{S}$ and the upper bound of linear combinations of ${\tilde{R}}_{S}$ about receivers 1–3, eliminating ${\tilde{R}}_{123}$, ${\tilde{R}}_{12}$, ${\tilde{R}}_{13}$, ${\tilde{R}}_{23}$ and ${\tilde{R}}_{1},{\tilde{R}}_{2},{\tilde{R}}_{3}$ by our elimination procedure yields the characterization (13a).

The procedure of the elimination for the case where *K*=3 is as follows, which is based on Lemma 3 that the sum of lower bounds for $\sum_{S\in J_{i}^{l}}r_{S},\;\forall i\in \left[N\right]$ is not greater than the lower bound for $\sum_{S\in J^{l}}r_{S}$ and observation (21) and (22) that shows the properties of the bounds derived from packing lemma. The detailed explanation for the procedure is elaborated in Section 5.2.5.

To start with, T={1,2,3} and $\pi_{\left\{1,2,3\right\}}=\left\{1,2,3\right\}$, as $R_{1}=R_{1,123}+R_{1,12}+R_{1,13}+R_{1,1},R_{2}=R_{2,123}+R_{2,12}+R_{2,23}+R_{2,2},R_{3}=R_{3,123}+R_{3,23}+R_{3,13}+R_{3,3}$ and $R_{123}≜R_{1,123}+R_{2,123}+R_{3,123}$, $R_{12}≜R_{1,12}+R_{2,12}$, $R_{13}≜R_{1,13}+R_{3,13}$, $R_{23}≜R_{2,23}+R_{3,23}$, $R_{1}≜R_{1,1}$, $R_{2}≜R_{2,2}$, $R_{3}≜R_{3,3}$. We can express $R_{1}+R_{2}+R_{3}$ as

$$\begin{array}{cc} & R_{1,1}+R_{1,12}+R_{1,13}+R_{1,123}\\ + & R_{2,2}+R_{2,12}+R_{2,23}+R_{2,123}\\ + & R_{3,3}+R_{3,13}+R_{3,23}+R_{3,123}, \end{array}$$

where with composing order $\pi_{\left\{1,2,3\right\}}=\left\{1,2,3\right\}$, $R_{1}+R_{2}+R_{3}$ can be expressed as $\left(R_{123}+R_{12}+R_{13}+R_{1}\right)+\left(R_{23}+R_{2}\right)+\left(R_{3}\right)$. The additives in the brackets are components from corresponding inequalities resulted from packing lemma, namely,

$$\begin{array}{cc} R_{1}+R_{2}+R_{3} & =\left(R_{123}+{\tilde{R}}_{12}+{\tilde{R}}_{13}+{\tilde{R}}_{1}\right)+\left({\tilde{R}}_{23}+{\tilde{R}}_{2}\right)+\left({\tilde{R}}_{3}\right), \end{array}$$

which is remained to combine the lower and upper bounds of the corresponding combination to derive one upper bound as shown in Equations (13g)–(13i).

Then, for T={1,2} and $\pi_{\left\{1,2\right\}}=\left\{1,2\right\}$, we can write $R_{1}+R_{2}$ as

$$\begin{array}{cc} & R_{1,1}+R_{1,12}+R_{1,13}+R_{1,123}\\ + & R_{2,2}+R_{2,12}+R_{2,23}+R_{2,123}, \end{array}$$

which is equal to $\left(R_{123}+{\tilde{R}}_{12}+{\tilde{R}}_{13}+{\tilde{R}}_{1}\right)+\left({\tilde{R}}_{23}+{\tilde{R}}_{2}\right)-\left(R_{3,123}+R_{3,13}+R_{3,23}\right)$ following the order $\pi_{\left\{1,2\right\}}=\left\{1,2\right\}$, where $\left(R_{3,123}+R_{3,13}+R_{3,23}\right)$ can be ignored as they are non-negative. Applying the corresponding bounds again, we obtain one from the Equation (13d).

For T={1} and $\pi_{\left\{1\right\}}=\left\{1\right\}$, by following the previous process we get Equation (13a). Thus, after consider ∀T⊆K and all permutations $\pi_{T}\in \Pi_{T}$, we get all inequalities in Equations (13a)–(13i).

### 5.2. Coding Scheme for K≥2

For each k∈K, split message $M_{k}\in \left[1:2^{nR_{k}}\right]$ into ∑j=1KKj−1 submessages $M_{k}≜\left(M_{k,S}:S\subseteq K,\;\left|S\right|=1,2,\cdots ,K\right)$ with $M_{k,S}\in \left[1:2^{R_{k,S}}\right]$ and $R_{k,S}=0$ if k∉S. Similarly to K=3 case, we use the following notations:

(17) $$\begin{array}{c} {\tilde{R}}_{S}≜\sum_{k=1}^{K}{\tilde{R}}_{k,S},\;R_{S}≜\sum_{k=1}^{K}R_{k,S},\;r_{S}≜{\tilde{R}}_{S}-R_{S}, \end{array}$$

and $M_{S}≜\left(M_{1,S},\cdots ,M_{K,S}\right)$. To simplify notations, we denote $U_{S}^{n}\left(m_{S},l_{S}|m_{A_{S}^{1:K}\backslash S},l_{A_{S}^{1:K}\backslash S}\right)$ by $U_{S}^{n}\left(m_{S},l_{S}\right)$ and its realization $u_{S}^{n}\left(m_{S},l_{S}|m_{A_{S}^{1:K}\backslash S},l_{A_{S}^{1:K}\backslash S}\right)$ by $u_{S}^{n}\left(m_{S},l_{S}\right)$.

#### 5.2.1. Codebook Generation

Fix a pmf $p(u\left(A^{1:K}\right))$ and function $x(u\left(A^{1:K}\right))$ and let ${\tilde{R}}_{S}\geq R_{S}$, $\forall S\in A^{1:K}$. Randomly and independently generate $2^{nR_{K}}$ sequences $u_{K}^{n}\left(m_{K}\right)$, $m_{K}\in \prod_{i=1}^{K}\left[1:2^{nR_{i,K}}\right]$ each according to $\prod_{i=1}^{n}p_{U_{K}}\left(u_{K,i}\right)$. For all $S\in A^{1:(K-1)}$, construct a subcodebook $C_{S}\left(m_{S}\right)$ consisting of $2^{n({\tilde{R}}_{S}-R_{S})}$ i.i.d. generated sequences $u_{S}^{n}\left(m_{S},j_{S}\right)$, $\left(m_{S},j_{S}\right)\in \prod_{i=1}^{K}\left[1:2^{nR_{i,S}}\right]\times \left[1:2^{n({\tilde{R}}_{S}-R_{S})}\right]$, each according to $\prod_{i=1}^{n}p_{U_{S}|U\left(A_{S}^{1:K}\backslash S\right)}\left(u_{S,i}|u_{S^{\prime },i}:S^{\prime }\in \left(A_{S}^{1:K}\backslash S\right)\right)$.

#### 5.2.2. Encoding

We iteratively find all indices $\left(j_{S}:S\in A^{1:K-1}\right)$ such that all selected codewords are jointly typical. In each round l=K−1,K−2,…,1, given messages $\left(m_{S},S\in A^{l}\right)$ and sequences $\left(u_{S}^{n}\left(m_{T},j_{T}\right):T\in A^{l+1:K-1}\right)$, find indices $\left(j_{S}:S\in A^{l}\right)$, such that all

$$\left(\left(u_{S}^{n}\left(m_{S},j_{S}\right):S\in A^{l}\right),\left(u_{S}^{n}\left(m_{T},j_{T}\right):T\in A^{l+1:K-1}\right),u_{K}^{n}\left(m_{K}\right)\right)\in T_{\epsilon_{1}}^{\left(n\right)}.$$

If there is more than one such tuple, pick an arbitrary one among them. If no such tuple exists, pick $(j_{S}:S\in A^{1:K})=1$. Then, generate $x^{n}\left(m_{S}:S\in A^{1:K}\right)$ with

$$x_{i}=x\left(u_{K,i}\left(m_{K}\right),\;u_{S,i}\left(m_{S},j_{S}\right):\forall S\in A^{1:(K-1)}\right),$$

for i∈[n].

To send the message tuple $\left(m_{1},\ldots ,m_{K}\right)=\left(m_{S}:S\in A^{1:K}\right)$, transmit $x^{n}\left(m_{S}:S\in A^{1:K}\right)$.

#### 5.2.3. Decoding

Let $\epsilon >\epsilon^{\prime }$. Decoder *k*, k∈K, declares that ${\hat{m}}_{k}$ is sent if it is the unique message such that

$$\left(y_{k}^{n},u_{S}^{n}\left(m_{S},j_{S}\right):S\in A^{1:(K-1)}\left(i\right),u_{K}^{n}\left(m_{K}\right)\right)\in T_{\epsilon }^{\left(n\right)},$$

for some $\left(u_{S}^{n}\left(m_{S},j_{S}\right):S\in A_{\left\{k\right\}}^{1:(K-1)},u_{K}^{n}\left(m_{K}\right)\right)$∈$\prod_{S\in A^{1:(K-1)}}C_{S}\left(1\right)\times C_{K}\left(1\right)$.

#### 5.2.4. Analysis of the Probability of Error

Assume without loss of generality that $(M_{1},\ldots ,M_{K})=1$ and let $(J_{S}:S\in A^{1:(K-1)}))=1$ be the index tuple of the chosen sequences $\left(U_{S}:S\in A^{1:(K-1)}\right)\in \prod_{S\in A^{1:(K-1)}}C_{S}\left(1\right)$. Then, decoder *i* makes an error only if one or more of the following events occur:

$$\begin{array}{cc} \; & E_{0}=\left\{\left(U_{S}^{n}\left(1,j_{S}\right):S\in A^{l:(K-1)},U_{K}^{n}\left(1\right)\right)\notin T_{\epsilon^{\prime }}^{\left(n\right)},\;\forall j_{S}\in \left[1:2^{nr_{S}}\right],\exists l\in \left[1:\left(K\;-\;1\right)\right]\right\},\\ \; & E_{k,1}=\left\{\left(Y_{k}^{n},U_{S}^{n}\left(1,1\right)\;:\;S\in A_{\left\{k\right\}}^{1:(K-1)},U_{K}^{n}\left(1\right)\right)\notin T_{\epsilon }^{\left(n\right)}\right\},\\ \; & E_{k,2}=\left\{\left(Y_{k}^{n},U_{S}^{n}\left(M_{S},J_{S}\right)\right)\in T_{\epsilon }^{\left(n\right)},\;\exists \;S\subseteq \left[1:K\right],M_{S}\neq 1\;\mathrm{or}\;\;J_{S}\neq 1,\right\}. \end{array}$$

Therefore, the probability of error for decoder *i* is upper bounded as

$$P\left(E_{k}\right)\leq P\left(E_{0}\right)+P\left(E_{0}^{c}\cap E_{k,1}\right)+P\left(E_{0}^{c}\cap E_{k,2}\right).$$

To bound $P(E_{0})$, we utilize the hierarchical covering lemma in Lemma 2. Therefore, $P(E_{0})$ tends to zero as n→∞ if

(18) $$\begin{array}{cc} \sum_{S\in J^{l}}r_{S}> & \sum_{S\in J^{l}}H\left(U_{S}|U\left(A_{S}^{1:K}\backslash S\right)\right)-H\left(U\left(J^{l}\right)|U\left(A^{(l+1):K}\right)\right)+\delta \left(\epsilon_{l}\right), \end{array}$$

holds for all l∈[1:(K−1)] and $J^{l}\in A^{l}$.

To bound $P(E_{0}^{c}\cap E_{k,1})$, by the conditional typicality lemma [2] it tends to zero as n→∞.

To bound $P(E_{0}^{c}\cap E_{k,2})$, note that $U_{S}^{n}\left(1,1\right)∼\prod_{i=1}^{n}$$p_{U_{S}|U\left(A_{S}^{1:K}\backslash S\right)}\left(u_{S,i}|u_{S^{\prime },i}\;\;:\;\;S^{\prime }\in A_{S}^{1:K}\backslash S\right)$ and $Y_{k}^{n}$ are independent of $U_{S}^{n}\left(M_{S},J_{S}\right)\notin C_{S}\left(1\right)$, $\forall S\in A_{\left\{k\right\}}^{1:(K-1)}$, as well as $U_{K}^{n}\left(M_{K}\right)\notin C_{K}\left(1\right)$. Furthermore, if $M_{T}\neq 1$ or $J_{T}\neq 1$ for any subset $T\in A_{\left\{k\right\}}^{1:K}$, then by the conditional coding distribution we have $U_{S}^{n}\left(M_{S},j_{S}\right)\notin C_{S}\left(1\right)$ for all $S\in A_{\left\{k\right\}}^{1:K}\left(T\right)$. Thus, ∀k∈K, $\forall J\subseteq A_{\left\{k\right\}}^{1:K}$ by identifying ${⋃}_{T\in J}A_{\left\{k\right\}}^{1:K}\left(T\right)$, we obtain inequalities with the help of packing lemma: for all $\forall J\subseteq A_{\left\{k\right\}}^{1:K}$,

(19) $$\begin{array}{cc} \; & \sum_{S\in {⋃}_{T\in J}A_{\left\{k\right\}}^{1:K}\left(T\right)}\left(\sum_{k=1}^{K}{\tilde{R}}_{k,S}+r_{S}\right)<\;\;\sum_{S\in {⋃}_{T\in J}A_{\left\{k\right\}}^{1:K}\left(T\right)}H\left(U_{S}|U\left(A_{S}^{1:K}\backslash S\right)\right)\\ \; & \;-H\left(U\left(\;\underset{S\in J}{⋃}\;A_{\left\{k\right\}}^{1:K}\left(S\right)\right)|Y_{k}U\left(A_{\left\{k\right\}}^{1:K}\backslash \;\underset{S\in J}{⋃}\;A_{\left\{k\right\}}^{1:K}\left(S\right)\right)\right)-\delta \left(\epsilon \right). \end{array}$$

#### 5.2.5. Eliminating $\left({\tilde{R}}_{k,S},r_{S}:k\in K,S\in A^{1:K}\right)$

Due to massive numbers of ${\tilde{R}}_{k,S}$ and $r_{S}$, using standard Fourier–Motzkin elimination to obtain the achievable rate region is disastrous. We first present observations which help the elimination, and then find all valid constraints for $\sum_{k\in T}R_{k}$ for all T⊆K.

**Observation**: Let $S\left(k,J\right)≜{⋃}_{T\in J}A_{\left\{k\right\}}^{1:K}\left(T\right)$, denote the right hand term of (19) by $I_{S(k,J)}$, and from (17), (19) can be rewritten as below.

(20) $$\begin{array}{cc} \; & \sum_{S\in S(k,J)}{\tilde{R}}_{S}<I_{S(k,J)}. \end{array}$$

(a) If there exists $k^{\prime },k^{\prime \prime }$ and $J^{\prime },J^{\prime \prime }$ such that

(21a) $$\begin{array}{c} S\left(k,J\right)=S\left(k^{\prime },J^{\prime }\right)\cup S\left(k^{\prime \prime },J^{\prime \prime }\right), \end{array}$$

then

(21b) $$\begin{array}{c} I_{S(k,J)}\leq I_{S{\left(k^{\prime },J\right)}^{\prime }}+I_{S(k^{\prime \prime },J^{\prime \prime })}. \end{array}$$

(b) If there exists $k^{\prime }$ and $J^{\prime }$ such that $S\left(k,J\right)\subseteq S\left(k^{\prime },J^{\prime }\right),$ then

(22) $$\begin{array}{c} I_{S(k,J)}\leq I_{S{\left(k^{\prime },J\right)}^{\prime }}. \end{array}$$

From the rate-splitting procedure, we have $R_{k}=\sum_{S\in A_{\left\{k\right\}}^{1:K}}R_{k,S}$, for all k∈K. From (17), (20) and the observations (21) and (22), we can obtain that the valid constraints for $R_{1},\ldots ,R_{K}$ will only have form $\sum_{k\in T}R_{k}$, i.e., any rate constraint $\sum_{j}a_{j}R_{j}$, for all $a_{j}\geq 0$ can be derived from $\sum_{k\in T}R_{k}$, for all T⊆K. Note that this will not hold for DM-BCs with *common* message (there exists an original source message to be sent to all receivers).

Thus, in order to find rate constraints for $\sum_{k\in T}R_{k}$, we must find all valid constraints for $\sum_{k\in T}\sum_{S\in A(k)}R_{k,S}$. By definitions in (17), we have

(23) $$\begin{array}{ccc} \sum_{k\in T}R_{k}=\sum_{k\in T}\sum_{S\subseteq K,k\in [|S|]}R_{k,S}=\sum_{S\in {⋃}_{k\in T}A_{\left\{k\right\}}^{1:K}}R_{S} & = & \sum_{S\in {⋃}_{k\in T}A_{\left\{k\right\}}^{1:K}}\;\;{\tilde{R}}_{S}-\;\;\sum_{S\in {⋃}_{k\in T}A_{\left\{k\right\}}^{1:K}}\;\;r_{S}. \end{array}$$

Now we show that how to obtain the closed-form rate region via set decomposition in (7) and the observations in Lemma 3, (21) and (22).

Consider a permutation $\pi_{T}\in \Pi_{T}$. For easy reading, from the decomposition (7), we have

(24) $$\begin{array}{c} \underset{k\in T}{⋃}A_{\left\{k\right\}}^{1:K}=\overset{\left|T\right|}{\underset{i=1}{⨆}}B_{\pi (i)}^{1:K}. \end{array}$$

For $R_{\pi (1)}$, it must involve $R_{\pi (1),S}:S\in A_{\pi (1)}^{1:K}$. Since $A_{\left\{\pi (1)\right\}}^{1:K}=B_{\pi (1)}^{1:K}=S\left(\pi \left(1\right),K\right)$ is the largest set with every element containing π(1), by observation in (21) and (22), we have the only valid rate constraint

$$\sum_{S\in A_{\pi (1)}^{1:K}}{\tilde{R}}_{S}\leq I_{A_{\left\{\pi (1)\right\}}^{1:K}}=I_{B_{\pi (1)}^{1:K}}$$

to support $R_{\pi (1)}$, where the last equality holds by (6).

For $R_{\pi (2)}$, $\sum_{S\in A^{1:K}\left(\pi \left(1\right)\right)}{\tilde{R}}_{S}$ already contains $\sum_{S\in A_{\left\{\pi (1)\pi (2)\right\}}^{1:K}}R_{k,S}$, thus we need to find the rate constraints for $\sum_{S\in A_{\pi (2)}^{1:K}\backslash A_{\left\{\pi (1),\pi (2)\right\}}^{1:K}}R_{k,S}$. Since $A_{\pi (2)}^{1:K}\backslash A_{\left\{\pi (1),\pi (2)\right\}}^{1:K}=B_{\pi (2)}^{1:K}=S\left(\pi (2),K\backslash \{\pi (1)\}\right)$, and $B_{\pi (2)}^{1:K}$ is second largest sets with every element containing π(2) and excluding π(1), by observation in (21) and (22), we obtain the only effective rate constraints

$$\sum_{S\in B_{\pi (2)}^{1:K}}{\tilde{R}}_{S}\leq I_{B_{\pi (2)}^{1:K}}$$

to support $R_{\pi (1)}$+$R_{\pi (2)}$. Iteratively applying the similar steps above and by (23), we find all valid rate constraints under the permutation π for $\sum_{k\in T}R_{k}$:

(25) $$\begin{array}{ccc} \sum_{k\in T}R_{k} & = & \sum_{S\in {⋃}_{k\in T}A_{\left\{k\right\}}^{1:K}}\;\;{\tilde{R}}_{S}-\;\;\sum_{S\in {⋃}_{k\in T}A_{\left\{k\right\}}^{1:K}}\;\;r_{S} \end{array}$$

(26) $$\begin{array}{ccc} & \overset{\left(a\right)}{\leq } & \sum_{i=1}^{\left|T\right|}I_{B_{\pi (i)}^{1:K}}-\sum_{S\in {⋃}_{k\in T}A_{\left\{k\right\}}^{1:K}}r_{S}, \end{array}$$

where (a) hods by (20) and $A_{\pi (i)}^{1:K}\backslash A_{\left\{\pi (1),\ldots ,\pi (i-1)\right\}}^{1:K}=B_{\pi (i)}^{1:K}=S\left(\pi (i),K\backslash \{\pi (1),\ldots ,\pi (i-1)\}\right)$.

Thus, formally we have for all T⊆K and $\pi \in \Pi_{T}$:

$$\begin{array}{cc} \;\;\sum_{k\in T}R_{k} & \;\leq \sum_{i=1}^{\left|T\right|}I_{B_{\pi (i)}^{1:K}}-\sum_{S\in {⋃}_{k\in T}A_{k}^{1:K}}r_{S}\\ \; & \overset{\left(a\right)}{=}\sum_{i=1}^{\left|T\right|}I_{B_{\pi (i)}^{1:K}}-\sum_{l=1}^{K-1}\sum_{S\in {⨆}_{i=1}^{\left|T\right|}B_{\pi (i)}^{l}}r_{S}\\ \; & \overset{\left(b\right)}{<}\sum_{i=1}^{\left|T\right|}(\sum_{S\in B_{\pi (i)}^{1:K}}H\left(U_{S}|U\left(A_{S}^{1:K}\backslash S\right)-H\left(U\left(B_{\pi (i)}^{1:K}\right)\;|\;Y_{\pi (i)}U\left(A_{\pi (i)}^{1:K}\;\;\backslash \;B_{\pi (i)}^{1:K}\right)\;\right)\;\right)\;\\ \; & \;+\sum_{l=1}^{K-1}\left(H\left(U\left(\overset{\left|T\right|}{\underset{i=1}{⨆}}B_{\pi (i)}^{l}\right)|U\left(A^{(l+1):K}\right)\right)-\;\sum_{S\in {⨆}_{i=1}^{\left|T\right|}B_{\pi (i)}^{l}}H\left(U_{S}|U\left(A_{S}^{1:K}\backslash S\right)\right)\right), \end{array}$$

where (*a*) comes from the disjoint decomposition in (7); (*b*) holds by letting $J^{l}={⨆}_{i=1}^{\left|T\right|}B_{\pi_{i}}^{l}$ in Lemma 2 and by Lemma 3.

## 6. Conclusions

In this paper, we propose new scheme for *K*-receiver DM-BCs with private messages based on exhaustive message splitting and *K*-level Marton’s coding. A hierarchical covering lemma is established which extends the 2-level multivariate covering lemma to *K*-level case. Our works provides a closed-form achievable rate region for the general *K*-user DM-BCs. In the future, we will find out some examples to justify that *K*-level Marton’s coding is strictly better than the 2-level one. 

## Figures and Tables

**Table 1:** Table of joint pmfs of all possible error messages. Symbol * denotes the message or index $m_{s}\neq 1_{s},l_{s}^{\prime }\neq l_{s},s\in \left\{123,12,13,1\right\}$, and $p^{*}$ denote joint pmf $p(u_{123}^{n},u_{12}^{n},u_{13}^{n},u_{1}^{n})$.

$m_{123}$	$\left(m_{12},l_{12}^{\prime }\right)$	$\left(m_{13},l_{13}^{\prime }\right)$	$m_{1,1}$	Joint pmf
1	$\left(1,l_{12}\right)$	$\left(1,l_{13}\right)$	1	$p^{*}p\left(y_{1}^{n}|u_{123}^{n}u_{12}^{n}u_{13}^{n}u_{1}^{n}\right)$
1	$\left(1,l_{12}\right)$	$\left(1,l_{13}\right)$	*	$p^{*}p\left(y_{1}^{n}|u_{123}^{n}u_{12}^{n}u_{13}^{n}\right)$
1	$\left(1,l_{12}\right)$	*	*	$p^{*}p\left(y_{1}^{n}|u_{123}^{n}u_{12}^{n}\right)$
1	*	$\left(1,l_{13}\right)$	*	$p^{*}p\left(y_{1}^{n}|u_{123}^{n}u_{13}^{n}\right)$
1	*	*	*	$p^{*}p\left(y_{1}^{n}|u_{123}^{n}\right)$
*	*	*	*	$p^{*}p\left(y_{1}^{n}\right)$

## Data Availability

Not applicable.

## Appendix A. Proof of Hierarchical Covering Lemma 2

Let

(A1) $$\begin{array}{ccc} M_{A^{l}} & ≜ & \prod_{S\in A^{l}}M_{S}=\prod_{S\in A^{l}}\left[1:2^{nr_{S}}\right]. \end{array}$$

Divide the messages and codewords into *K* levels, where at level l∈[1:K], there are messages sets $\left\{M_{S}:S\in A^{l}\right\}$, and codewords

$$\left(U_{S}^{n}\left(m_{S}\right):S\in A^{l},m_{S}\in M_{A^{l}}\right).$$

The proof of hierarchical covering lemma starts at level l=K−1, finding a message tuple $\left(m_{S}:S\in A^{K-1}\right)$ such that $\left(u_{S}^{n}\left(m_{S}\right):S\in A^{K-1},u_{K}^{n}\right)\in T_{\epsilon_{K-1}}^{\left(n\right)}$. Then, with a descending order of l=K−2,K−1,…,1, we find a message tuple $\left(m_{S}:S\in A^{l}\right)$ such that $\left(\left(u_{S}^{n}\left(m_{S}\right):S\in A^{l}\right),u_{K}^{n}\right)\in T_{\epsilon_{l}}^{\left(n\right)}$.

For each l∈[1:K], we use $S_{i}^{l}$ to denote the element of $A^{l}$ for $i=1,\ldots ,|A^{l}|$, i.e.,

$$A^{l}=\left\{S_{1}^{l},\ldots ,S_{|A^{l}|}^{l}\right\}.$$

We define, for all l∈[1:K] , the random events

$$\begin{array}{ccc} E_{l} & = & \left\{\left(\left(U_{S}^{n}\left(m_{S}\right):S\in A^{l:(K-1)}\right),U_{K}^{n}\right)\notin T_{\epsilon_{l}}^{\left(n\right)},\;\;\forall \;\left(m_{S}:S\in A^{l:(K-1)}\right)\in \;\;\prod_{S\in A^{l:(K-1)}}\;\;\left[1:2^{nr_{S}}\right]\right\},\\ A_{l} & = & \{\left(m_{S_{1}^{l}},\ldots ,m_{S_{|A^{l}|}^{l}}\right)\in M_{A^{l}}:\\ & & \;\left(U^{n}\left(m_{S_{1}^{l}}\right)\ldots U^{n}\left(m_{S_{|A^{l}|}^{l}}\right),u^{n}\left(A^{l+1:K}\right)\right)\in T_{\epsilon_{l}}^{\left(n\right)},|u^{n}\left(A^{l+1:K}\right)\in T_{\epsilon_{l+1}}^{\left(n\right)}\},\\ Q_{l} & = & \left(u^{n}\left(m_{S_{1}^{j}}\right)\ldots u^{n}\left(m_{S_{|A^{j}|}^{j}}\right)\;:\;j\in \left[\left(l+1\right)\;:\;K\right]\right)\in T_{\epsilon_{l}}^{\left(n\right)}, \end{array}$$

where

$$u^{n}\left(A^{l+1:K}\right)=\left(u^{n}\left(m_{S_{1}^{j}}\right)\ldots u^{n}\left(m_{S_{|A^{j}|}^{j}}\right)\;:\;j\in \left[\left(l+1\right)\;:\;K\right]\right).$$

Note that $E_{l}$ denotes the event that there isn’t any tuple of sequences from level *l* to *K* such that they are jointly typical. Correspondingly, $E_{l}^{c}$ denotes the event that there exists such tuple of sequences. $A_{l}$ denotes the set of messages at level *l* such that the corresponding codewords are jointly typical with a given tuple of jointly typical codewords from levels l+1 to *K*. $Q_{l}$ denotes a condition that there exists message $\left(m_{S_{1}^{l}},\ldots ,m_{S_{|A^{l}|}^{l}}\right)\in M_{A^{l}}$ at level l+1,...,K such that the corresponding codewords are jointly typical.

Our goal is to find rate conditions such that there exists a tuple of messages $\left(m_{S}:S\in A^{1:K}\right)$ satisfying $\left(U_{S}^{n}\left(m_{S}\right):S\in A\right)\in T_{\epsilon_{1}}^{\left(n\right)}$, which is equivalent to $P\{E_{1}\}\to 0$ as n→∞.

The probability of $E_{1}$ can be upper bounded as

$$\begin{array}{ccc} P\{E_{1}\} & = & P\left\{E_{1}\cap E_{2}^{c}\right\}+P\left\{E_{1}\cap E_{2}\right\}\\ & \leq & P\left\{E_{1}|E_{2}^{c}\right\}+P\left\{E_{2}\right\}. \end{array}$$

With recursively upper bounding $P(E_{l})$, we obtain

(A2) $$\begin{array}{ccc} P\{E_{1}\} & \leq & \sum_{l=1}^{K-1}P\left\{E_{l}|E_{l+1}^{c}\right\}+P\left\{E_{K}\right\}\\ & = & \sum_{l=1}^{K-1}P\left\{A_{l}\right\}+P\left\{E_{K}\right\}, \end{array}$$

where the last equality holds by definitions of $A_{l}$ and $E_{l}$. As $P(E_{K})$ tends to zero as n→∞ by the property of typicality, and *K* is a fixed channel parameter, in order to make $P\{E_{1}\}$ tend to 0, it is sufficient to let $P\{A_{l}\}$ tend 0 as n→∞ for all l∈[1:K−1].

To analyze (A2), $P(E_{K})$ tends to zero as n→∞ by the condition of the lemma. Each term $P\{E_{l}|E_{l+1}^{c}\}$ is equivalent to $P\left\{|A_{l}|=0\right\}$, which is upper bounded by a variation of Chebyshev inequality:

$$P\left\{|A_{l}|=0\right\}\;\leq \;P\left\{{\left(|A_{l}\left|-E\right|A_{l}|\right)}^{2}\;\leq \;{\left(E|A_{l}|\right)}^{2}\right\}\;\leq \;\frac{\mathrm{Var}\left(|A_{l}|\right)}{{\left(E|A_{l}|\right)}^{2}}.$$

Using indicator random variables, $|A_{l}|$ can be written as

(A3a) $$\begin{array}{ccc} |A_{l}| & = & \sum_{j=1}^{|A^{l}|}\sum_{m_{S_{j}^{l}}\in M_{S_{j}^{l}}}I\left(m_{S_{1}^{l}},\ldots ,m_{S_{|A^{l}|}^{l}}\right) \end{array}$$

where

(A3b) =I(mS1l,…,mS|Al|l)=1ifQlgivenQl+1,0otherwise,

for each $\left(m_{S_{1}^{l}},\ldots ,m_{S_{|A^{l}|}^{l}}\right)\in M_{A^{l}}$.

For all $J^{l}\subseteq A^{l}$, define

(A4) $$\begin{array}{ccc} p_{J^{l}}=P\{ & & \left(U^{n}\left(m_{S_{1}^{l}}\right)\ldots U^{n}\left(m_{S_{|A^{l}|}^{l}}\right),u^{n}\left(A^{l+1:K}\right)\right)\in T_{\epsilon_{l}}^{\left(n\right)},\\ & & \left(U^{n}\left(m_{S_{1}^{l}}^{\prime }\right)\ldots U^{n}\left(m_{S_{|A^{l}|}^{l}}^{\prime }\right),u^{n}\left(A^{l+1:K}\right)\right)\in T_{\epsilon_{l}}^{\left(n\right)},\\ & & \;\mathrm{with}\;m_{S_{j}^{l}}=m_{S_{j}^{l}}^{\prime }\;\mathrm{if}\;S_{j}^{l}\in J^{l}\;\mathrm{and}\;\mathrm{vice}\;\mathrm{versa}|u^{n}\left(A^{l+1:K}\right)\in T_{\epsilon_{l+1}}^{\left(n\right)}\}. \end{array}$$

From (A3) and definition of $p_{J^{l}}$ in (A4), we have

$$\begin{array}{ccc} E\left(|A_{l}|\right) & = & 2^{n\sum_{S\in A^{l}}r_{S}}\cdot p_{A^{l}},\\ E\left(|A_{l}{|}^{2}\right) & = & \sum_{m_{S_{1}^{l}},\ldots ,m_{S_{\;|A^{l}|}^{l}}}\sum_{\begin{array}{c} m_{S}^{\prime }\neq m_{S},\\ \forall S\in A^{l}\backslash J^{l} \end{array}}p_{J^{l}}.\\ & \leq & 2^{n\sum_{S\in A^{l}}r_{S}}\cdot \sum_{J^{l}\subseteq A^{l}}2^{n\sum_{S\in A^{l}\backslash J^{l}}r_{S}}\cdot p_{J^{l}}. \end{array}$$

From definition of $p_{J^{l}}$ and independence of codewords, we have ${\left(p_{A_{l}}\right)}^{2}=p_{\emptyset }$. Therefore,

$$\mathrm{Var}(|A_{l}\left|\right)\leq 2^{n\sum_{S\in A^{l}}r_{S}}\sum_{J^{l}\subseteq \left\{A^{l}\backslash \emptyset \right\}}2^{n\sum_{S\in A^{l}\backslash J^{l}}r_{S}}\cdot p_{J^{l}}.$$

Next, we compute the upper bound of $p_{J^{l}}$.

(A5) $$\begin{array}{ccc} p_{J^{l}}=\; & & \;P\{\left(U^{n}\left(m_{S_{1}^{l}}\right)\ldots U^{n}\left(m_{S_{|A^{l}|}^{l}}\right),u^{n}\left(A^{l+1:K}\right)\right)\in T_{\epsilon_{l}}^{\left(n\right)},\\ & & \;\left(U^{n}\left(m_{S_{1}^{l}}^{\prime }\right)\ldots U^{n}\left(m_{S_{|A^{l}|}^{l}}^{\prime }\right),u^{n}\left(A^{l+1:K}\right)\right)\in T_{\epsilon_{l}}^{\left(n\right)},\\ & & \;\;\mathrm{with}\;m_{S_{j}^{l}}=m_{S_{j}^{l}}^{\prime }\;\mathrm{if}\;S_{j}^{l}\in J_{l}\;\mathrm{and}\;\mathrm{vice}\;\mathrm{versa}|Q_{l+1}\}\\ =\; & & \;P\left\{\;\left(U^{n}\left(m_{S_{1}^{l}}\;\right)\ldots U^{n}\left(m_{S_{\;\;|A^{l}|}^{l}}\;\right),\;u^{n}\left(A^{l\;+\;1:K}\right)\right)\;\in \;T_{\epsilon_{l}}^{\left(n\right)}\;|\;Q_{l\;+\;1}\right\}\\ & & \;\cdot \;P\{\left(U^{n}\left(m_{S_{1}^{l}}^{\prime }\right)\ldots U^{n}\left(m_{S_{\;|A^{l}|}^{l}}^{\prime }\right),u^{n}\left(A^{l+1:K}\right)\right)\in T_{\epsilon_{l}}^{\left(n\right)}\\ & & \;\;\;\;|\;\;\left(U^{n}\left(m_{S_{1}^{l}}\;\right)\ldots U^{n}\left(m_{S_{\;|A^{l}|}^{l}}\;\;\right),\;u^{n}\left(A^{l\;+\;1:K}\right)\right)\;\in \;T_{\epsilon_{l}}^{\left(n\right)},\;Q_{l\;+\;1}\}\\ \overset{\left(a\right)}{\leq }\; & & \;2^{n\left(H\left(U\left(A^{l}\right)|U\left(A^{l+1:K}\right)\right)+\delta \left(\epsilon_{l}\right))\right)}\cdot \;2^{-n(\sum_{S\in A^{l}}\;\;H\left(U_{S}|U\left(A_{S}^{1:K}\backslash S\right)-\delta \left(\epsilon_{l}\right)\right)}\\ & & \;\cdot \;2^{n\left(H\left(U\left(A^{l}\backslash J^{l}\right)|U\left(J^{l}\right),U(A^{(l+1):K}\right)\right)+\delta \left(\epsilon_{l}\right))}\cdot \;2^{-n\left(\sum_{S\in A^{l}\backslash J^{l}}H\left(U_{S}|U\left(A_{S}^{1:K}\backslash S\right)\right)-\delta \left(\epsilon_{l}\right)\right)} \end{array}$$

where (*a*) follows by properties of joint typicality and the mutually conditional independence property, i.e., given $u^{n}\left(A^{(l+1):K}\right)$, $\left(U_{S}^{n}\left(m_{S}^{\prime }\right):S\in A^{l}\backslash J^{l}\right)$ and $\left(U_{S}^{n}\left(m_{S}\right):S\in A^{l}\backslash J^{l}\right)$ are conditionally independent. The lower bound of $p_{A^{l}}$ can be derived as

(A6) $$\begin{array}{ccc} p_{A^{l}}\; & & =P\left\{\left(U^{n}\left(m_{S_{1}^{l}}\right)\ldots U^{n}\left(m_{S_{|A^{l}|}^{l}}\right),u^{n}\left(A^{l+1:K}\right)\right)\in T_{\epsilon_{l}}^{\left(n\right)},|u^{n}\left(A^{l+1:K}\right)\in T_{\epsilon_{l+1}}^{\left(n\right)}\right\},\\ & & \geq \left(1-\epsilon_{l}\right)\cdot 2^{n\left(H\left(U\left(A^{l}\right)|U\left(A^{l+1:K}\right)\right)-\delta \left(\epsilon_{l}\right)\right))\;}\cdot \;2^{-n\left(\sum_{S\in A^{l}}H\left(U_{S}|U\left(A_{S}^{1:K}\backslash S\right)\right)+\delta \left(\epsilon_{l}\right)\right)}. \end{array}$$

where the last equality holds by the properties of jointly typicality. From (A5) and (A6), we have

$$\begin{array}{ccc} \frac{p_{J^{l}}}{{\left(p_{A^{l}}\right)}^{2}}\leq & & \;\;2^{n\left(H\left(U\left(A^{l}\right)|U\left(A^{l+1:K}\right)\right)+\delta \left(\epsilon_{l}\right))\right)}\cdot \;2^{-n(\sum_{S\in A^{l}}H\left(U_{S}|U\left(A_{S}^{1:K}\backslash S\right)-\delta \left(\epsilon_{l}\right)\right)}\\ & & \;\cdot \;2^{n\left(H\left(U\left(A^{l}\backslash J^{l}\right)|U\left(J^{l}\right),U\left(A^{(l+1):K}\right)\right)+\delta \left(\epsilon_{l}\right)\right)}\cdot \;{\left(1-\epsilon_{l}\right)}^{-2}\cdot 2^{2n\left(H\left(U\left(A^{l}\right)|U\left(A^{l+1:K}\right)\right)+\delta \left(\epsilon_{l}\right))\right)\;}\\ & & \;\cdot \;2^{-2n(\sum_{S\in A^{l}}H\left(U_{S}|U\left(A_{S}^{1:K}\backslash S\right)\right)+\delta \left(\epsilon_{l}\right)})\\ \leq & & \;\;{\left(1-\epsilon_{l}\right)}^{-2}\cdot 2^{-nH(U\left(A^{l}\right)|U\left(A^{(l+1):K}\right))}\cdot \;2^{n(\sum_{S\in A^{l}}H\left(U_{S}|U\left(A_{S}^{1:K}\backslash S\right)\right)}\\ & & \;\cdot \;2^{n(H\left(U\left(A^{l}\backslash J^{l}\right)|U\left(J^{l}\right)U\left(A^{(l+1):K}\right)\right)}\cdot \;2^{-n\sum_{S\in A^{l}\backslash J^{l}}H\left(U_{S}|U\left(A_{S}^{1:K}\backslash S\right)\right)}\cdot 2^{n\delta (\epsilon )}. \end{array}$$

Therefore, we finally obtain the desired inequality

=Var(|Al|)(E(|Al|))2≤(1−ϵ)−2·2−n∑S∈AlrS·∑Jl⊆Al\∅2n∑S∈Al\JlrS·pJl(pAl)2≤(1−ϵ)−2∑Jl⊆Al\∅(2−n∑S∈JlrS·2nδ(ϵ)=·2nH(U(Al\Jl)∣U(Jl)U(A(l+1):K))−H(U(Al)∣U(A(l+1):K))=·2n(∑S∈AlH(US∣U(AS1:K\S))−∑S∈Al\JlH(US∣U(AS1:K\S)))),

which tends to zero as n→∞ if for all $J^{l}\subseteq A^{l}$

(A7) $$\begin{array}{ccc} \sum_{S\in J^{l}}r_{S}> & & \;H\left(U\left(A^{l}\backslash J^{l}\right)|U\left(J^{l}\right),U\left(A^{(l+1):K}\right)\right)-\;H\left(U\left(A^{l}\right)|U\left(A^{(l+1):K}\right)\right)\\ & & \;+\;\sum_{S\in A^{l}}H\left(U_{S}|U\left(A_{S}^{1:K}\backslash S\right)\right)-\;\sum_{S\in A^{l}\backslash J^{l}}H\left(U_{S}|U\left(A_{S}^{1:K}\backslash S\right)\right)+\delta \left(\epsilon \right)\\ = & & \;\;H\left(U\left(A^{l}\backslash J^{l}\right)|U\left(J^{l}\right),U\left(A^{(l+1):K}\right)\right)-\;H\left(U\left(A^{l}\right)|U\left(A^{(l+1):K}\right)\right)\\ & & \;+\;\sum_{S\in J^{l}}H\left(U_{S}|U\left(A_{S}^{1:K}\backslash S\right)\right)+\delta \left(\epsilon \right)\\ = & & \;\sum_{S\in J^{l}}H\left(U_{S}|U\left(A_{S}^{1:K}\backslash S\right)\right)-\;H\left(U\left(J^{l}\right)|U\left(A^{(l+1):K}\right)\right)+\delta \left(\epsilon \right). \end{array}$$

Therefore, from (A2), $P\{E_{1}\}$ tends to zero as n→∞ if conditions (A7) hold for all l∈[1:(K−1)].
